# Neuromodulation for Intractable Pain

**DOI:** 10.3390/brainsci10050267

**Published:** 2020-04-30

**Authors:** Alexander L. Green, Tipu Z. Aziz

**Affiliations:** Nuffield Department of Surgical Sciences, University of Oxford, Oxford OX3 9DU, UK; tipu.aziz@nds.ox.ac.uk

Over 7% of the Western population suffer from intractable pain and despite pharmacotherapy, many patients’ pain is refractory [1]. In addition to the pain, patients often suffer from depression and anxiety, poor quality of life and loss of employment. An ever-enlarging problem is that of opiate use, which in the US has been labelled as a “crisis” [2]. In order to tackle these issues, we require a greater understanding of the underlying pathophysiology of pain, novel treatments (pharmacological and otherwise), and a greater evidence base for both the efficacy of non-pharmacological treatments alongside a better understanding of the mechanisms of action. In this issue, Deer et al. [3] provide an up-to-date literature review on spinal cord stimulation (SCS), dorsal root ganglion (DRG) stimulation, and peripheral nerve stimulation (PNS), which are all well-established neuromodulatory techniques for treating chronic neuropathic pain. Deer et al. provide a comprehensive report, demonstrating that SCS has well-established efficacy for specific pain subtypes such as failed back surgery syndrome (FBSS), complex regional pain syndrome (CRPS), and a number of other conditions. They point out that although SCS is not a new therapy, there are a multitude of new advancements in the field such as novel waveforms, new closed-loop technologies, and many recent advances in the understanding of its mechanisms. Whilst DRG stimulation and PNS are somewhat more recent additions to the armamentarium, there is good early evidence for efficacy, although the authors point out that trial designs (especially subject blinding) can be a challenge. Dones and Levi, in their review of SCS, echo the conclusions of Deer et al. and also discuss in depth the technical nuances of SCS therapy. Controversies include the choice between percutaneous and paddle electrodes, and the choice between awake implantation and implantation under general anaesthetic. The authors present the evidence on different sides of the argument, providing the advantages and disadvantages of each technique. This also makes the point that trials need to be evaluated in the context of the specific technique. Regarding the mechanisms of action of DRG stimulation, Parker et al. [4] report a study in which magnetoencephalography (MEG) was used to measure cortical activity during periods of DRG stimulation compared with a control whilst performing a cognitive task (the “N-Back task”). The authors elegantly show that DRG stimulation modulates cortical gamma activity in the cognitive dimension of pain. This study has implications for the way in which peripheral neuromodulation works and implies that the modulation of cortical networks is important (either as a cause or consequence), and not just local DRG effects. Salgado et al. [5], in their study on CRPS in mice, bring to our attention that there are alternatives to medication, other than neuromodulation. One such intervention is manual therapy such as ankle joint mobilization. The authors show that mobilization 48 hours after an ischemia–reperfusion injury reduced the pain behaviour and oxidative stress. This study outlines the importance of therapy in the acute phase after injury in order to prevent the build-up of chronic pain in the first place.

For those patients who do not respond to SCS and other forms of more “peripheral” neuromodulation, deep brain stimulation (DBS) and motor cortex stimulation (MCS) are alternatives. Farrell and colleagues [6] review the history and literature on these treatments and conclude that whilst there are many studies showing efficacy, there is a lack of well-designed clinical trials and that more work is needed to assess the factors that predict success in individual patients. Farrell et al. also summarise a newer target for DBS for pain: the anterior cingulate cortex (ACC). Further work on ACC DBS for chronic pain is highlighted by Huang et al [7]. Their study follows an individual who gained successful pain relief with bilateral ACC DBS but unfortunately also developed disabling generalised seizures that were related to the stimulation amplitude. By applying a novel brain recording device (Medtronic PC + S®, Minneapolis, MN, USA), the authors were able to identify the patterns of stimulation that precluded the seizure activity. This is a prime example of how evolution in device technology can enable successful treatment in patients that have been deemed “untreatable” with existing technology. In a second study, Farrell et al. [8] highlight the use of DBS for a range of pain and non-pain conditions. The latter concentrates mainly on autonomic symptoms such as hypertension and bladder symptoms, often investigated in the context of DBS for existing conditions such as Parkinson’s disease. The authors point out that DBS is a useful treatment for a range of chronic symptoms that cause suffering and that the realm of palliative care is not just for patients with a limited life expectancy. 

In addition to studies looking at neuromodulation as a general treatment for refractory conditions, more work is needed into its use in specific pain syndromes. Roy et al. [9] summarise pelvic and urogenital pain and the use of neuromodulation in its management. The authors demonstrate that the neurocircuitry underpinning the pelvic and urogenital system may be targeted from peripheral (e.g., posterior tibial or pudendal nerves) to central (periaqueductal grey area). Again, there are many gaps in our knowledge regarding both mechanisms of action and efficacy. There is also much more work needed to understand the underlying molecular changes in pain sub-types that will help inform drug design but also influence the targets for neuromodulation. Lombardo et al. present an intriguing study looking at the interleukin-1 receptor antagonist (IL-1RN) expression in a murine cortical spreading depression (CSD) model of migraine [10]. The investigators demonstrate that there is an upregulation of IL-1RN and hypothesise that this demonstrates a possible attempt to modulate the inflammatory response. The link between chronic pain and the immune system is gaining increasing interest in the literature and it is likely that further investigation is important for both chronic pain management and the tentative possibility of using neuromodulation to alter the immune response, as is already being investigated in relation to vagal nerve stimulation [11].

## References

[1] Torrance N., Smith B.H., Bennett M.I., Lee A.J. (2006). The Epidemiology of Chronic Pain of Predominantly Neuropathic Origin. Results From a General Population Survey. J. Pain.

[2] Ostling P.S., Davidson K.S., Anyama B.O., Helander E.M., Wyche M.Q., Kaye A.D. (2018). America’s Opioid Epidemic: A Comprehensive Review and Look into the Rising Crisis. Curr. Pain Headache Rep..

[3] Deer T., Jain S., Hunter C., Chakravarthy K.V. (2019). Neurostimulation for Intractable Chronic Pain. Brain Sci..

[4] Parker T., Huang Y., Raghu A.L., Fitzgerald J.J., Green A.L., Aziz T.Z. (2020). Dorsal Root Ganglion Stimulation Modulates Cortical Gamma Activity in the Cognitive Dimension of Chronic Pain. Brain Sci..

[5] Salgado A.S.I., Stramosk J., Ludtke D.D., Kuci A.C.C., Salm D.C., Ceci L.A., Petronilho F., Florentino D., Danielski L.G., Gassenferth A. (2019). Manual Therapy Reduces Pain Behavior and Oxidative Stress in a Murine Model of Complex Regional Pain Syndrome Type I. Brain Sci..

[6] Farrell S.M., Green A., Aziz T. (2018). The Current State of Deep Brain Stimulation for Chronic Pain and Its Context in Other Forms of Neuromodulation. Brain Sci..

[7] Huang Y., Cheeran B., Green A.L., Denison T.J., Aziz T.Z. (2019). Applying a Sensing-Enabled System for Ensuring Safe Anterior Cingulate Deep Brain Stimulation for Pain. Brain Sci..

[8] Farrell S.M., Green A.L., Aziz T.Z. (2019). The Use of Neuromodulation for Symptom Management. Brain Sci..

[9] Roy H., Offiah I., Dua A. (2018). Neuromodulation for Pelvic and Urogenital Pain. Brain Sci..

[10] Lombardo S.D., Mazzon E., Basile M., Cavalli E., Bramanti P., Nania R., Fagone P., Nicoletti F., Petralia M. (2019). Upregulation of IL-1 Receptor Antagonist in a Mouse Model of Migraine. Brain Sci..

[11] Huffman W.J., Subramaniyan S., Rodriguiz R.M., Wetsel W., Grill W.M., Terrando N. (2019). Modulation of neuroinflammation and memory dysfunction using percutaneous vagus nerve stimulation in mice. Brain Stimul..

